# Clarithromycin synergizes with cisplatin to inhibit ovarian cancer growth in vitro and in vivo

**DOI:** 10.1186/s13048-019-0570-9

**Published:** 2019-11-08

**Authors:** Bo Zhou, Meng Xia, Bin Wang, Niresh Thapa, Lijuan Gan, Chaoyang Sun, Ensong Guo, Jia Huang, Yulan Lu, Hongbin Cai

**Affiliations:** 1 Department of Gynecologic Oncology, Zhongnan Hospital of Wuhan University, 169 Donghu Road, Wuhan, 430071 Hubei Province China; 2 Hubei Key Laboratory of Tumor Biological Behaviors, Wuhan, People’s Republic of China; 3 Hubei Cancer Clinical Study Center, Wuhan, People’s Republic of China; 4 Cancer Biology Research Center, Tongji Hospital, Tongji Medical College, Huazhong University of Science and Technology, Wuhan, 430030 Hubei China; 5 Hubei Provincial Institute for Food Supervision and Test, Wuhan, 430075 China; 6 Karnali Academy of Health Sciences, Jumla, Nepal

**Keywords:** Ovarian cancer, Clarithromycin, Cisplatin-resistance, Combination therapy

## Abstract

**Background:**

Cisplatin-based chemotherapy is the first-line treatment for ovarian cancer. However, acquired resistance to cisplatin treatment often occurs in epithelial ovarian cancer, and effective and practical methods for overcoming this obstacle are urgently needed. The study aimed to demonstrate the synergistic effect of clarithromycin (CAM) with cisplatin to inhibit ovarian carcinoma cells growth in vitro and in vivo.

**Results:**

We performed CCK-8 assay to detect apoptosis rates in response to CAM alone or in combination with cisplatin, which were further confirmed by Annexin V and PI staining methods and western blotting. Mechanistically, CAM could reduce endogenous antioxidant enzyme expression and increase the levels of reactive oxygen species (ROS) to augment the cytotoxic effect of cisplatin. Meanwhile, a tumor xenograft model in athymic BALB/c-nude mice demonstrated that CAM combined with cisplatin resulted in reduced tumor growth and weight compared with cisplatin alone.

**Conclusion:**

Collectively, our results indicate that CAM works synergistically with cisplatin to inhibit ovarian cancer cell growth, which may be manipulated by a ROS-mediated mechanism that enhances cisplatin therapy, and offers a novel strategy for overcoming cisplatin therapy resistance.

## Background

Ovarian cancer is the fourth most common cancer among women and the most lethal gynecologic malignancy worldwide. It was estimated that 22,440 new cases were diagnosed and approximately 14,080 died from this disease in 2017 in the United States [1]. Currently, cytoreductive surgical debulking accompanied by platinum- or paclitaxel-based chemotherapy is considered to be the first-line therapy for advanced ovarian cancer. Although the majority of patients initially respond well to cisplatin (DDP)-based treatment, they later develop resistance against the treatment [2]. The mechanisms underlying DDP resistance in ovarian cancer have not been fully clarified. Thus, there is an urgent need to explore the molecular mechanisms and develop novel strategies to overcome this dilemma.

DDP, used as a single agent or in combination with other anti-neoplastic agents, is an important chemotherapeutic drug for the treatment of various cancers, including ovarian carcinoma. DDP induces cell cycle arrest and apoptosis by forming both intra- and interstrand crosslinks in the DNA to inhibit replication and transcription [3]. Numerous studies have indicated that the therapeutic effect of DDP or other anticancer agents occurs by enhancing the generation of reactive oxygen species (ROS), which are primarily produced in mitochondria [4, 5].

Clarithromycin (CAM) belongs to a family of 14-membered ring macrolide antibiotics that has been used worldwide since 2005. Recently, extensive preclinical and clinical data suggest that CAM plays a potential role in treating various tumors when combined with conventional chemotherapy drugs, including DDP, cyclophosphamide and adriamycin. Based on previous studies, the principal mechanisms of anti-tumor activity are involved in pro-inflammatory cytokines or chemokines reduction [6], autophage attenuation [7], endoplasmic reticulum stress-mediated C/EBP homologous protein (CHOP) induction [8], anti-angiogenesis [9], nuclear transcription factors and reactive oxygen species et al. [10]. However, the mechanisms underlying anti-tumor activity remain unclear and are poorly understood.

In this study, we show that CAM not only increases anti-tumor effects in a dose-dependent manner, but it can also serve as an adjuvant that displays synergistic effects with DDP to enhance the cytotoxicity in ovarian cancer cells. These effects involve in increasing ROS generation by reducing the expressions of endogenous antioxidant enzymes. Taken together, DDP chemotherapy in combination with the conditional cost-efficient drugs CAM may provide a novel strategy for ovarian cancer treatment.

## Materials and methods

### Cell culture

A DDP-resistant human epithelial ovarian cancer cell line C13* was a gift from Prof. Benjamin K. Tsang at the Ottawa Health Research Institute, Ottawa, Canada [11]. The ovarian cancer cell line SKOV3 was purchased from the American Type Culture Collection (Manassas, VA, USA). Cells were cultured in Macoy’5A medium (Gibco, Gaithersburg, MD, USA) supplemented with 10% fetal bovine serum (Gibco, Gaithersburg, MD, USA), penicillin (100 units/mL), and streptomycin (100 μg/mL) at 37 °C in a humidified atmosphere containing 5% CO_2_.

### Reagents and antibodies

Clarithromycin (CAM) was purchased from Selleck Chemicals (Houston, TX, USA); it was dissolved in dimethyl sulfoxide (DMSO). DDP was obtained from Sigma-Aldrich (St. Louis, MO, USA); it was dissolved in sterile filtered RNA-free water. N-acetyl cysteine (NAC) was purchased from Beyotime Biotechnology (Shanghai, China). Antibodies used for analysis included the following: primary rabbit polyclonal antibody against human cleaved-PARP (Epitomics, Burlingame, CA, USA), rabbit monoclonal antibody against human activated caspase-3 (Cell Signaling Technology, Inc., Beverly, MA, USA), rabbit monoclonal antibody against human SOD2 (Proteintech, Wuhan, China), goat monoclonal antibody against human 8-OHdG (Millipore, Bedford, MA, USA), and mouse monoclonal antibody against human γ-H2AX (Millipore, Bedford, MA, USA). The secondary antibody for GAPDH was obtained from Santa Cruze Biotech (Santa Cruz, CA, USA).

### Cell viability analysis

Cell viability was measured using a Cell Counting Kit-8 (Dojindo, Tokyo, Japan). Firstly, 5 × 10^3^ cells were seeded in a 96-well plate in triplicate and incubated for 24 h. Then, cells were treated with CAM and DDP separately or combined at 37 °C in a humidified atmosphere containing 5% CO_2_ for another 48 h. Next, a 1:10 diluted CCK-8 solution in Macoy’s 5A medium was added to cells, followed by incubation for 2 or 4 h at 37 °C in a humidified atmosphere containing 5% CO_2_. The absorbance was detected at 450 nm by a microplate reader (Bio-Rad Laboratories, Hercules, CA, USA). Each experiment was performed three times. The results were analyzed using GraphPad Prism 5(GraphPad Software Inc., San Diego, CA, USA). The combination index (CI) value was calculated with CompuSyn software for the combined treatment of CAM and DDP [12].

### Annexin V-FITC/PI staining analysis

Cells were treated with CAM and DDP separately or combined at indicated concentrations for 48 h, harvested with trypsin, and centrifuged for 5 min at 800 rpm. Then, cells were washed with phosphate-buffered saline (PBS) 3 times for 5 min each time. Cells were centrifuged again, and the supernatant was discarded. Next, cells were stained with Annexin V-FITC/PI according to the manufacturer’s instructions (KeyGen Biotech, Nanjing, Inc. China). Each experiment was performed three times.

### ROS measurement

ROS levels were measured using a Cellular Reactive Oxygen Species Detection Assay Kit (Abcam, Cambridge, MA, USA) according to the manufacturer’s instructions. Cells were stained with DCFH-DA for 20 min at 37 °C, then washed with PBS, trypsinized, centrifuged, and resuspended, and the cell density was adjusted to the range of 1.0 × 10^6^ ~ 2.0 × 10^7^. The fluorescence signal of DCF was obtained by FACSCalibur and analyzed using the CellQuest Software (Becton Dickinson, Mountain View, CA, USA).

### Mitotracker red analysis

Live C13* cells were prepared in a sticky-slide 8 well (Millipore Bedford, MA, USA) for 12 h, and then wells were treated with CAM and DDP separately or combined for 48 h. Cells were incubated in 0.5 μM mitoTracker Red diluted with the dye-working solution (Abcam, Cambridge, MA, USA) and incubated at 37 °C with 5% CO_2_ for 30 min. After staining, the cells were washed with culture medium two or three times and immediately detected through a fluorescence microscope (Olympus, Tokyo, Japan). The fluorescence intensity was calculated using Image J software.

### Western blotting

Cells were seeded in a 6-cm culture plate for 12 h and treated with CAM and DDP separately or combined for 48 h. Then cells were collected and lysed with RIPA lysis buffer. Cellular proteins were quantified using a DC Protein Assay Kit (Bio-Rad, Richmond, CA, USA). Next, 50 μg of the extracted proteins were loaded onto a 10% SDS-PAGE gel and transferred onto a PVDF membrane (Millipore Corp., Bedford, MA, USA)). First, the membrane was blocked with 5% BSA for 1 h and then incubated overnight at 4 °C with primary antibodies including rabbit monoclonal antibody against human activated caspase-3 (1:1000 dilution; Cell Signaling Technology, Inc., Beverly, MA, USA), rabbit polyclonal antibody against human cleaved-PARP (1:1000 dilution; Cell Signaling Technology, Inc., Beverly, MA, USA), and rabbit monoclonal antibody against human SOD2 (1:800 dilution; Proteintech Group, Inc., Wuhan, China). GAPDH was used as an internal control (rabbit monoclonal antibody against human GAPDH, 1:2000; Cell Signaling Technology, Inc., Beverly, MA, USA). Chemiluminescence was detected using an imaging system (Bio-Rad, Hercules, CA, USA).

### RNA extraction and real-time RT-PCR

Total RNA was extracted from ovarian cancer cells treated with CAM or DDP at indicated concentrations using the PrimeScript RT Reagent Kit (TaKaRa, Kusatsu, Japan) and SYBR Premix Ex Taq (TaKaRa, Kusatsu, Japan) according to the manufacturer’s instructions. Primer sequences for the endogenous reference gene, glyceraldehyde-3-phosphate dehydrogenase (GAPDH), and the specific primer sequences used are shown in Additional file 1: Table S1. The comparative Ct method was used to calculate the relative changes in gene expressions.

### In vivo xenograft studies

Four week-old female nude BALB/c mice (HFK Bioscience, Beijing, China) were subcutaneously injected with 5 × 10^6^ C13* cells resuspended in 100 μL PBS, in the right flank. Mice were housed in laminar flow cabinets under specific pathogen-free conditions and all animals’ experiments were carried out in accordance with the Guide for the Care and Use of Laboratory Animals of Zhongnan Hospital of Wuhan University and in accordance with the Guidelines on the Use of Laboratory Animals of the National Institutes of Health. When tumors reached a mean size of 50 mm^3^, the mice were randomly assigned into four groups with 6 mice per group (control, CAM, DDP, and combined groups). The DDP group was treated with 5 mg/kg DDP intraperitoneally once a week for 4 weeks. The CAM group was treated with 100 mg/kg CAM intraperitoneally once a day for four consecutive weeks. The combined group was treated with the above dosages for 4 weeks. The control group was injected with sterile saline. Tumor volumes were calculated as (length × width^2^)/ 2 every 4 days. The tumor weight was weighed when mice were sacrificed by cervical dislocation under anesthesia.

### Immunohistochemical staining and analysis

Immunohistochemical staining was performed as previously described [13]. The sections were stained with primary antibody anti-SOD2 (1:50) and anti-8OHdG (1:100), anti-γ-H2AX (1:1000), and anti-activated caspase3 (1:50), and IgG staining was used as a negative control. Positive 8-OHdG, γ-H2AX and active caspase3 staining were mainly detected in the nuclei, while positive SOD2 expression was detected primarily in the cytoplasm. Sample immunoreactivity was semi-quantitatively evaluated using the following criteria: strong positive (scored as 3), strong staining intensity (> 90% of cancer cells); moderate positive (2), moderate staining intensity (> 50–89% of positive cells); weak positive (1), weak staining intensity (> 10–49% of positive cells); absent (0), no staining intensity and few to no positive cells [14]. The intensity and proportion of staining in each section were calculated by two independent investigators using six random high-power field images for each group.

### Statistical analysis

All expressions analyses were performed in triplicate. All data are expressed as the mean ± SD. The statistical significance of differences was analyzed by two-tailed Student’s *t*-test or one-way ANOVA. A *P* value less than 0.05 was considered statistically significant. All statistical analyses were done using SPSS 21.0 (SPSS Inc., Chicago, IL).

## Results

Effect of CAM and DDP on ovarian cancer cell viability.

**Fig. 1:**
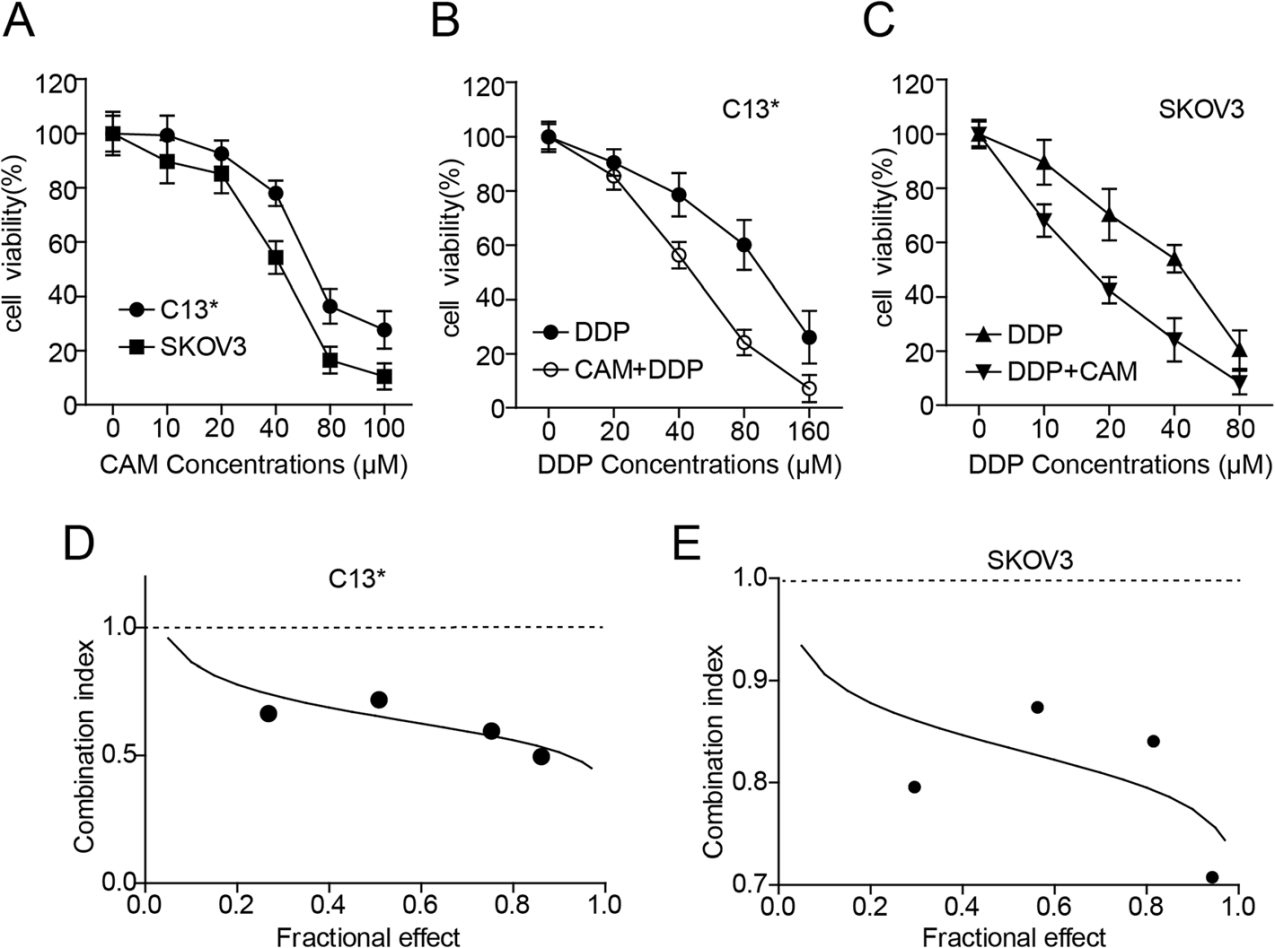
The effect of CAM alone or combined with DDP on ovarian cancer cells. **a** C13* and SKOV3 cells were treated with different concentrations of CAM for 48 h, and cell viability was assessed by CCK-8. **b** and **c** CCK-8 assay indicated that cell viability was significantly reduced in the CAM plus DDP group compared to the group treated with DDP alone in C13* and SKOV3 cells treated with different concentrations for 48 h. The concentration of CAM used in C13* and SKOV3 cells were 66 μM and 44 μM separately. **d** and **e** The combination index (CI) was used to calculate the synergistic effects displayed by the two-drug combination. The data indicated that CAM and DDP synergistically inhibited the growth of C13* and SKOV3 cells. CI values below 1.0 represented synergistic interactions of the two drugs

We hypothesized that CAM could exert an anti-neoplastic effect in ovarian cancer cells. Two ovarian cancer cell lines C13* and SKOV3 were used to assess the effect of CAM on cell viability via CCK-8 assay. After exposure to various concentrations of CAM for 48 h, we found that the cell viability of C13* and SKOV3 was reduced. The IC50 of CAM on C13* cells was approximately 66 μM (IC50 = 65.59 μM, 95% CI = 59.13–72.76 μM), whereas that of the cell viability of CAM on SKOV3 was up to 44 μM (IC50 = 43.87 μM, 95% CI = 35.79–53.78 μM) (Fig.1a). Next, we treated the two cell lines with the combination of the two drugs. Using the same method, we tested the cell viability of C13* and SKOV3 cells treated with DDP alone and DDP plus CAM. We found that the cell viability rates were significantly reduced in the combination group compared to the DDP group. The IC50 of C13* cells treated with DDP alone was decreased from approximately 100 μM to 46 μM when combined with CAM administration (DDP alone: IC50 = 98.46 μM, 95% CI = 66.19–146.5 μM; DDP plus CAM: IC50 = 45.50 μM, 95% CI = 42.68–48.52 μM). Meanwhile, similar results were observed in SKOV3 cells (DDP alone: IC50 = 39.86 μM, 95% CI = 22.01–72.19 μM; DDP plus CAM: IC50 = 16.84 μM, 95% CI = 13.44–21.11 μM) (Fig. 1b and c). To assess the synergetic or antagonistic effects of the two drug combination, we treated C13* and SKOV3 cells with various concentrations of CAM and DDP separately or combined at a fixed ratio of 1:1, as shown in Fig. 1d and e. The combination index (CI) calculated using CalcuSyn software was less than 1.0, which indicated that the two drugs had a synergetic effect.

CAM enhanced the cytotoxic effect of DDP and the apoptosis rate in ovarian cancer cells.

**Fig. 2:**
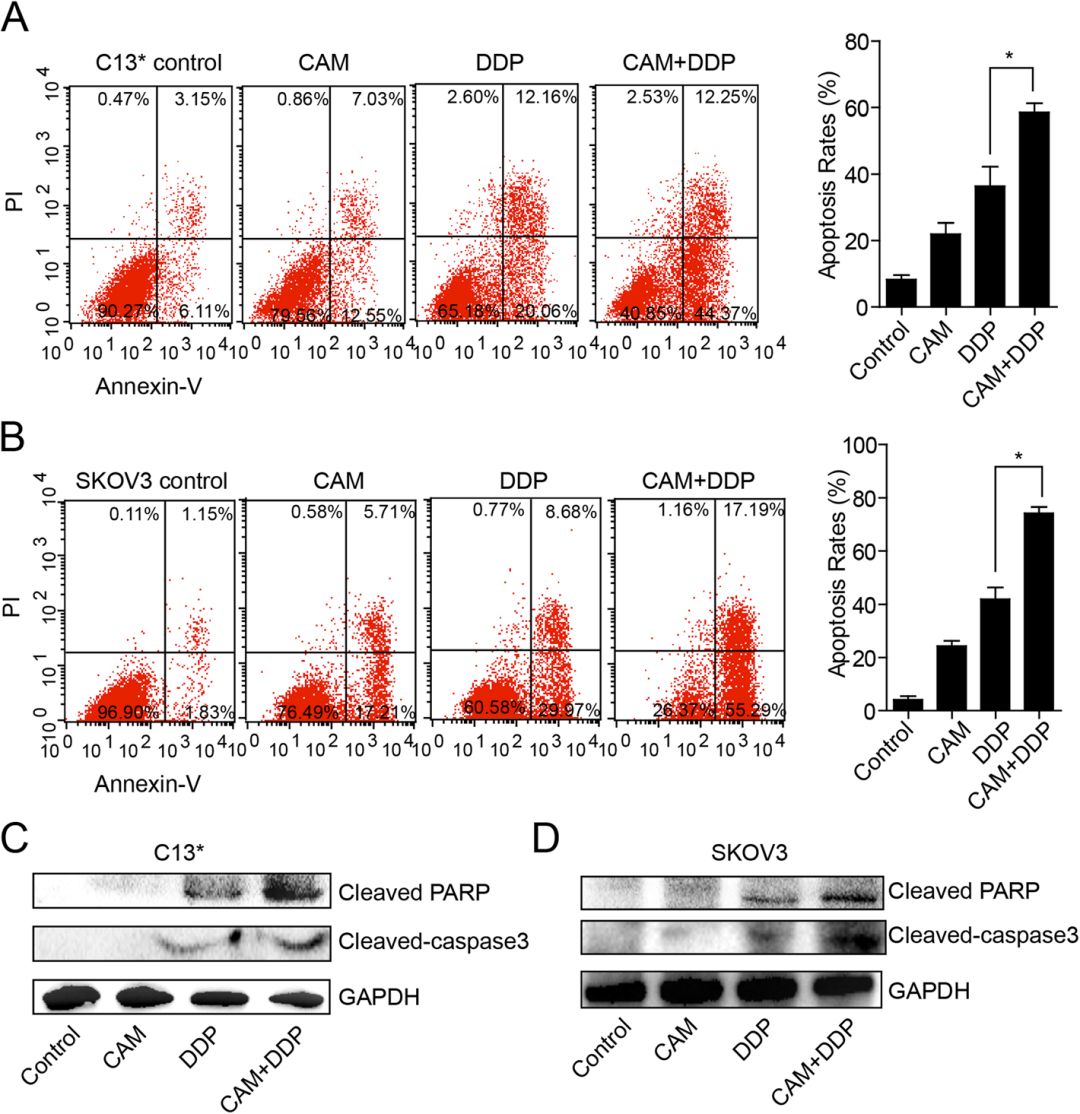
DDP combined with CAM synergistically promoted tumors cells apoptosis in C13* and SKOV3 cells. **a** C13* cells were treated with DDP (80 μM), CAM (20 μM) or both for 48 h, and Annexin V/PI staining was used to detect apoptosis rates. **b** SKOV3 cells were treated with DDP (40 μM), CAM (10 μM) or both for 48 h, and Annexin V/PI staining was used to detect apoptosis rates. **c** and **d** The expressions of cleaved PARP and activated caspase-3 were detected in C13* and SKOV3 cells treated with DDP, CAM or both for 72 h by western blotting analysis, respectively. GAPDH was used as a loading control. Each experiment was performed in triplicate. * *P* < 0.05

To further confirm the effects of the two drugs on apoptosis, C13* and SKOV3 cells were treated with 80 μM or 40 μM DDP, 20 μM or 10 μM CAM, or a combination of these, respectively, for 36 h. Then, cells were stained with Annexin V-FITC/PI and analyzed using flow cytometry to detect apoptosis. We found that the apoptosis rate was significantly increased in the combination group compared to DDP group, and this difference was statistically significant (Fig. 2a and b). Meanwhile, western blotting showed that the apoptosis-associated protein markers, cleaved PARP and cleaved caspase-3 were increased in the combination group compared with those in the DDP group (Fig. 2c and d). Collectively, these data suggested that the combination of CAM and DDP had a synergetic effect on ovarian cancer treatment via activation of the apoptotic signaling pathway. This result aligns with a previous report in human cervical cancer HeLa cells that involved activation of the mitochondria-mediated apoptotic signaling pathway following CAM treatment [15].

CAM acts synergistically with DDP with DDP to enhance mitochondria biogenesis and reduce endogenous antioxidant enzyme expression.

**Fig. 3:**
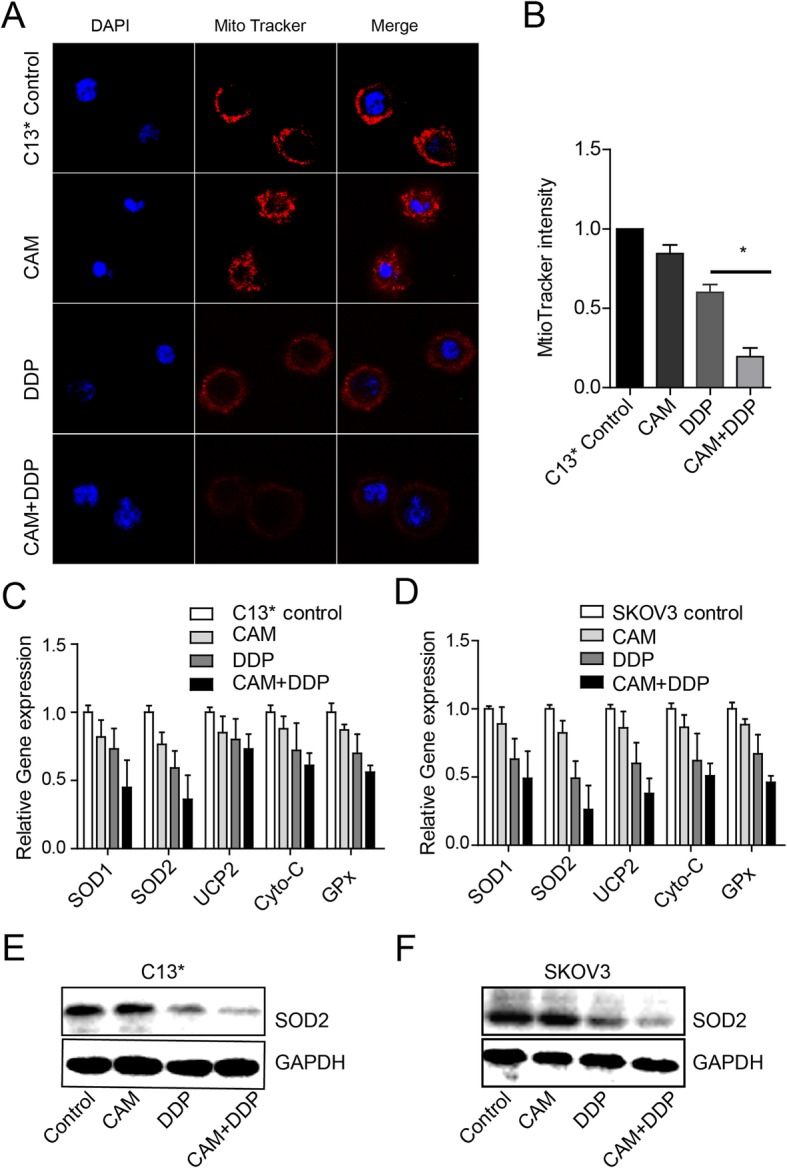
CAM increased mitochondrial biogenesis and down-regulated the expression of endogenous antioxidant enzyme expressions in combination with DDP in ovarian cancer cells. **a** and **b** C13* cells were treated with DDP, CAM or both, and mitotracker staining was used to track the quantity of mitochondria. Mitotracker staining was much weaker with combined treatment than with DDP or CAM alone. **c** and **d** The expression of endogenous antioxidant enzyme SOD1, SOD2, UCP2, Cyto-C, and GPx were detected by qRT-PCR in C13* and SKOV3 cells, respectively. **e** and **f** The expression of the key endogenous antioxidant enzyme SOD2 was detected through qRT-PCR in C13* and SKOV3 cells, respectively. Each experiment was performed in triplicate. * *P* < 0.05

Previous studies have demonstrated that CAM may have a profound impact on the anti-inflammatory or immunomodulatory properties that are involved in the mechanism responsible for generating ROS [16]. We hypothesized that CAM may impact mitochondria-derived generation ROS. We performed mito-tracker staining to track and measure the quantity of mitochondria. Firstly, C13* cells were treated with 50 μM DDP and 20 μM CAM for 48 h. As shown in Fig. 3a and b, the mitotracker intensity in the combined group was significantly weaker than in the other three treatment groups. This phenomenon could partly explain the impact of CAM on mitochondria biogenesis, which is known as the primary method of antioxidant enzyme production. Then, qRT-PCR method was performed to detect the expression of the antioxidant proteins SOD1, SOD2, UCP2, GPx, and Cyto-C. As illustrated in Fig. 3c and d, the expression of the key antioxidant enzyme SOD2 was decreased in the DDP plus CAM group compared with the DDP group, and these results were further confirmed in C13* and SKOV3 cells using western blotting (Fig. 3e and f).

### CAM increased ROS levels in ovarian cancer cells

**Fig. 4:**
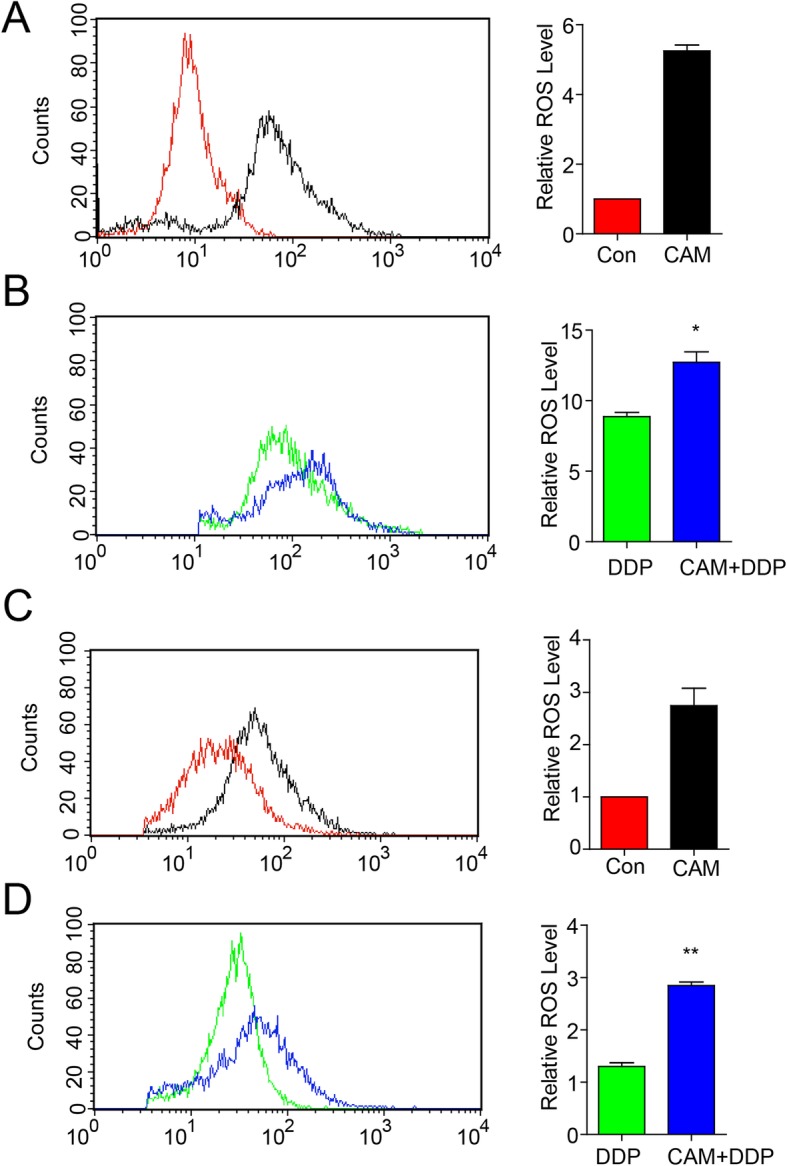
Measurement of intracellular ROS levels. **a** and **b** Intracellular ROS levels were determined by ROS assay in C13* cells treated with DDP with or without CAM. **c** and **d** Intracellular ROS levels were determined by ROS assay in SKOV3 cells treated with DDP with or without CAM. Flow cytometry analysis showed that the combined group had higher ROS generation than groups with DDP or CAM treatment alone. * *P* < 0.05, * *P < 0.005

**Fig. 5:**
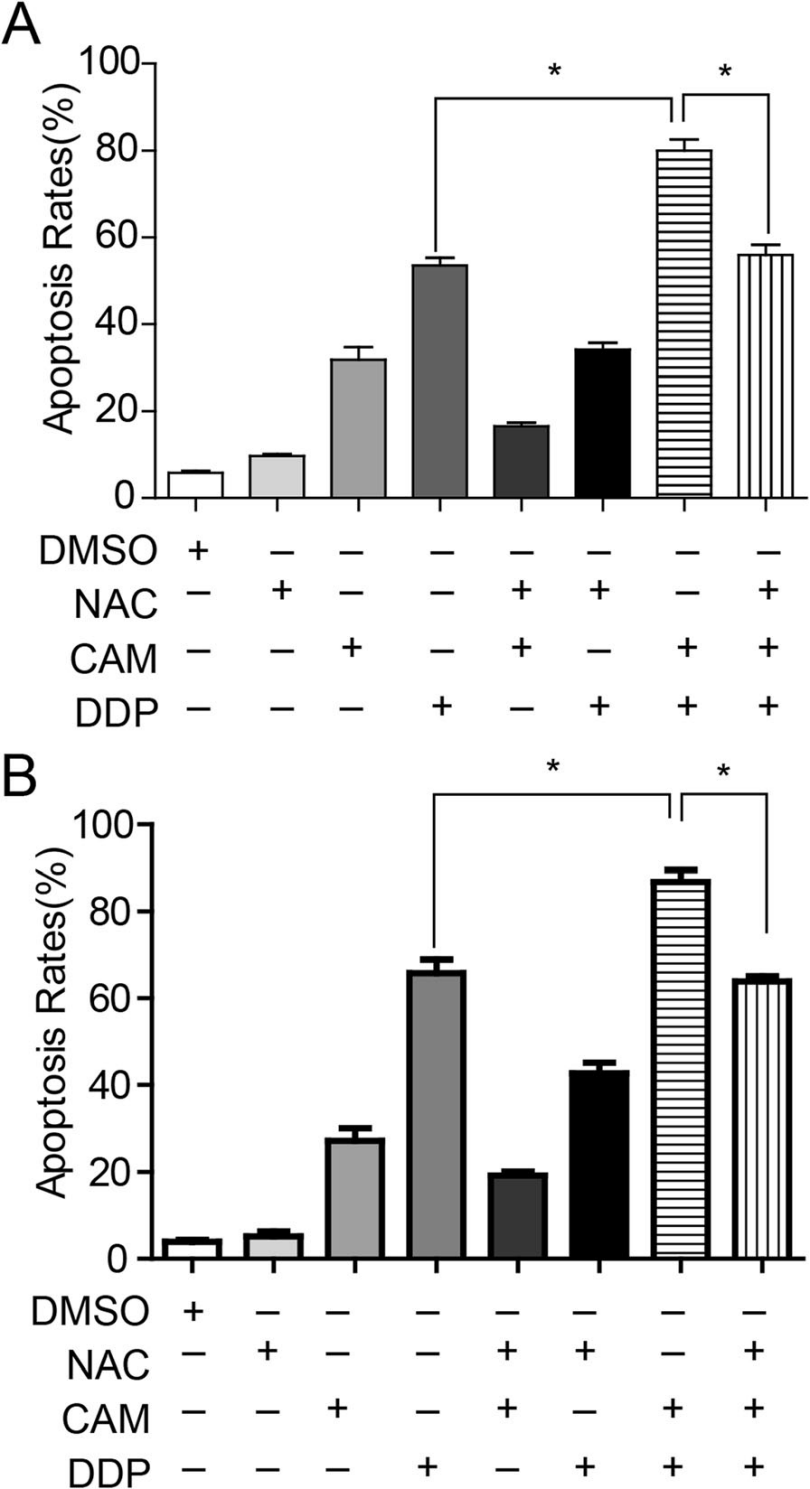
CAM-induced apoptosis was associated with endogenous ROS levels generated within mitochondria. **a** The antioxidant NAC was used to reduce the endogenous ROS levels. C13* cells were treated with DMSO, CAM (20 μM), DDP (80 μM), NAC(10 μM) and the combination of the drugs for 48 h, and apoptosis rates were detected by Annexin V/PI staining. **b** SKOV3 cells were treated with DMSO, CAM (10 μM), DDP (40 μM), NAC (10 μM), and the combination of the drugs for 48 h, and the apoptosis rates were detected by Annexin V/PI staining. Each experiment was performed in triplicate. * *P* < 0.05

Previous research has demonstrated that chemotherapeutic anti-cancer agents such as DDP and taxol, as well as the most efficient frame-shifting mutagens promoted or induced apoptosis through the generation of ROS mainly produced in mitochondria [17, 18]. To determine whether CAM plus DDP increased apoptosis in tumor cells by increasing intracellular ROS levels, ROS were measured using DCFH-DA staining. Compared with DDP treatment alone, the combined group displayed increased ROS levels that were up to 1.5-fold higher in C13* cells (*P* = 0.0404) (Fig. 4a and b), and 2.2-fold higher in SKOV3 cells (*P* = 0.004) (Fig. 4c and d). To further confirm whether CAM synergized with DDP to inhibit ovarian cancer cell growth by increasing ROS generation, the antioxidant NAC was used to reduce the intracellular ROS levels. As shown in Fig. 5a and b, Annexin V-FITC/PI staining showed increased rates of apoptosis in C13* and SKOV3 cells in response to combined treatment compared to DDP alone, whereas apoptosis was significantly inhibited by NAC administration. Collectively, these data at least suggested that CAM and DDP synergistically promoted ovarian cancer apoptosis by generating ROS synthesis.

CAM synergized with DDP to inhibit ovarian tumor growth in vivo*.*

**Fig. 6:**
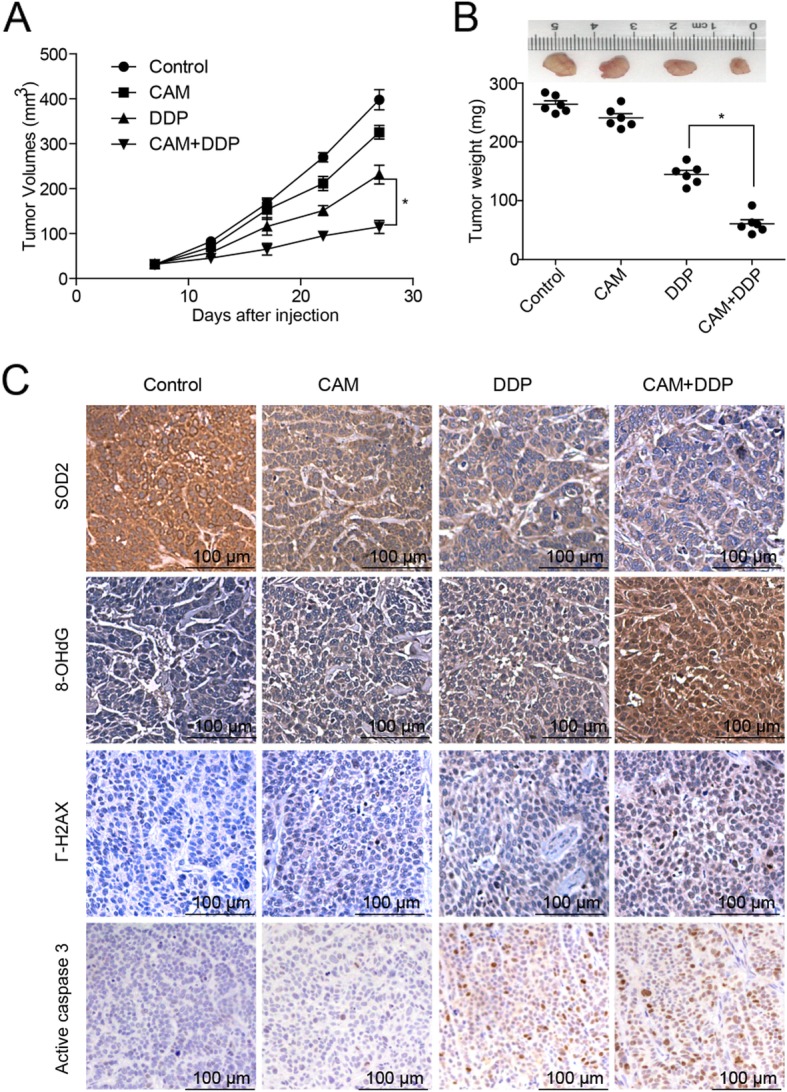
CAM and DDP synergistically inhibited ovarian tumor growth in vivo. First, 5 × 10^6^ C13* cells were subcutaneously injected into the right flank of female BALB/c-nude mice. Tumor volumes were calculated as length × width^2^/2 every four days with a caliper, and tumor weights were measured when mice were sacrificed by cervical dislocation under anesthesia. Mice were treated with 5 mg/kg DDP intraperitoneally per week for four weeks, 100 mg/kg CAM intraperitoneally every day for four weeks, or a combination of each. **a** The tumor volume was measured in each group. (*N* = 6 per group, DDP vs DDP plus CAM, *P* < 0.05). **b** The tumor weight was measured in each group. The tumor weight in the DDP plus CAM group was significantly lower than that in the group with DDP alone. (DDP vs DDP plus CAM, *P* < 0.05). **c** IHC analysis indicated that the expression of antioxidant enzyme, SOD2 was abundantly reduced, and marked increases were determined in the expression of the DNA damage proteins 8-OhdG and γ-H2AX, and the apoptotic protein activated caspase-3. The IHC score is calculated in Additional file 2: Figure S1

To further validate our findings in vivo, a xenograft model was established. C13* cells were injected into the right flank of BALB/c-nude mice. The tumor weight and tumor volume were assessed after the various drug treatments were administered for 4 weeks. The tumor volumes and weights in the combined group were smaller than those in the other three groups (Fig. 6a and b). We next performed immunohistochemical analysis to detect the expression levels of ROS-injury-associated proteins and the apoptosis associated proteins. IHC analysis indicated that as compared to the other three groups, the combined group exhibited significantly decreased expression of the antioxidant enzyme, SOD2, and displayed increased expression of γ-H2AX and 8-OHdG, which are markers of DNA damage and ROS injury, respectively. Meanwhile, compared to the tumors treated with the vehicle, DDP, or CAM alone, tumors treated with CAM plus DDP exhibited highly overexpressed activated caspase-3 (Fig. 6c). The IHC score of each gene is calculated in Additional file 1: Figure S1. Taken together, these results partly explained how CAM and DDP exert synergistic effects to promote apoptosis in ovarian tumors in that they down-regulated antioxidant enzyme expression and up-regulated intracellular ROS levels.

## Discussion

Ovarian cancer is the most lethal gynecologic cancer among women in the developed world. DDP-based chemotherapy remains the first-line treatment for this type of cancer. Although the majority of patients are initially sensitive to DDP chemotherapy, nearly 80–90% of those patients will eventually develop resistance to this treatment, leading to acquired DDP resistance and causing high mortality. Therefore, molecular targeted therapies or common low-cost drugs that can be combined with conventional treatments are urgently needed.

CAM is a well-known macrolide antibiotic traditionally used for various types of bacterial infections. Recently, extensive research has demonstrated that CAM may act as an anti-inflammatory agent rather than an antibacterial one [19]. Moreover, CAM may not only play a role in the treatment of various tumors, but it may also potentiate the antitumor activity of many kinds of cytotoxic drugs as well in vitro and in vivo [8, 9, 20]. In the present study, we showed that CAM treatment alone could induce apoptosis in ovarian cancer cells, attenuate ovarian tumor growth and function synergistically with DDP to potentiate antitumor activity. In vitro, we observed that the apoptosis rates of the two ovarian cancer cell lines were increased after CAM administration in a dose-dependent manner. In addition, the apoptosis rates were significantly increased when CAM was administered in combination with a conventional chemotherapy agent, DDP. In vivo, a subcutaneous xenograft model indicated that CAM could slightly inhibit the tumor growth, but this finding was not statistically significant (CAM group vs vehicle, *P* = 0.1145); however, the tumor weight was lower than that of the vehicle group, which was a statistically significant difference (CAM group vs vehicle, *P* = 0.0309). This conflict may be due to measuring error while recording each tumor size. More notably, the tumor volumes and weights were significantly lower after combined treatment than with DDP treatment alone, and this result was statistically significant. Mechanistically, we found that treatment with CAM and DDP led to increased ROS levels, which were mainly generated in mitochondria, due to decreased expression of intracellular antioxidant enzymes. This phenomenon was validated both in vitro and in vivo. Previous studies have demonstrated that multiple mechanisms may underlie the antitumor activity of CAM, and no consistent conclusion has been reached. A study performed by Qiao Ai-Min et al. demonstrated that CAM-induced apoptosis in human cervical cancer HeLa cells was activated through the mitochondrial-mediated apoptotic pathway that is involved in cytochrome c release and the activation of caspase-9 and caspase-3 [15]. Peng YC et al. reported that CAM administration could inhibit the NF-κB pathway in gastric epithelial cells, as NF-κB is a critical transcription factor that regulates inflammatory responses and innate immunity [21]. In non-small-cell lung cancer (NSCLC), Mikasa et al. found that NSCLC patients who received CAM treatment (200 mg b.i.d.) showed better conditions and prolonged survival times compared to those not treated with CAM due to reduced serum IL-6 levels after treatment [22, 23]. Aside from influencing secretory factors and cytokines, CAM affects the VEGF pathway [9]. Other research has suggested that antitumor activity in NSCLC patients might be partly mediated via the modulation of ERK1/2 by CAM administration [24]. Miki Nakamura et al. demonstrated that CAM augmented the antitumor activity of thalidomide against multiple myeloma (MM) cells through autophagy attenuation [7], and it has been reported that CAM enhanced bortezomib-induced cytotoxicity via ER stress-mediated CHOP induction in breast cancer cells [8]. ROS-mediated induction of apoptosis and protection from radiation-induced lung injury were also reported [10, 16]. To the best of our knowledge, ours is the first study to investigate the synergistic effects of CAM with DDP to promote the apoptosis of ovarian neoplasms, which was mediated by inducing an increase in ROS in vitro and in vivo.

Our study demonstrated for the first time that CAM augments DDP response when administered in combination via an ROS-mediated synergistic effect. However, this study still has several limitations. Other chemotherapeutic agents such as taxol have not been included in our current study. Moreover, in our study, CAM was administered simultaneously to induce tumor cell apoptosis, and whether treatment before or after chemotherapy affords similar effects has yet to be studied. Thus, further study investigating the use of this drug combination and role of different mechanisms in vitro and in vivo are greatly warranted.

## Conclusions

The combination of CAM and DDP interacted effectively and synergistically in the treatment of ovarian tumors. These findings demonstrated that CAM, as a low cost, low toxicity, commonly prescribed antibiotic, presents a novel strategy to treat ovarian cancer cells in combination with conventional chemotherapy, and should be considered for further investigation in clinical trials.

## Supplementary information

**Additional file 1 Table S1.** Primers used in this study for real-time PCR experiments. (DOC 38 kb)

**Additional file 2.** The IHC score of each gene is calculated as described in materials and methods (Fig. A,B,C and D). (TIF 461 kb)

## Data Availability

None.

## Abbreviations

95% CI: 95% confidence interval
CAM: Clarithromycin
CCK-8: Cell Counting Kit-8
CHOP: C/EBP homologous protein.
CI: Combination index
DDP: cisplatin
IC50: Half maximal inhibitory concentration
IL-6: Interleukin 6
NAC: N-acetyl-cysteine
PBS: Phosphate-buffered saline
ROS: Reactive oxygen species
