# Supplementary material for: Clarithromycin synergizes with cisplatin to inhibit ovarian cancer growth in vitro and in vivo
Source: J Ovarian Res. 2019 Nov 8;12:107. doi: 10.1186/s13048-019-0570-9 (PMC6839134; doi:10.1186/s13048-019-0570-9)
Supplement: Supplementary file 1 — Additional file 1 Table S1. Primers used in this study for real-time PCR experiments. (DOC 38 kb) [file 13048_2019_570_MOESM1_ESM.doc]

Supplementary Table1. Primers used in this study for real-time PCR experiments.

| Name | Primer | Sequence (5'->3') | Tm |
| --- | --- | --- | --- |
| SOD1 | Forward primer | TGGTTTGCGTCGTAGTCTCC | 60.04 |
| SOD1 | Reverse primer | CCAAGTCTCCAACATGCCTCT | 60.00 |
| SOD2 | Forward primer | GCACTAGCAGCATGTTGAGC | 59.90 |
| SOD2 | Reverse primer | GGGCTGTAACATCTCCCTTGG | 60.41 |
| cyto-c | Forward primer | CGGGGTGCCTTTAGGATTCA | 55.00 |
| cyto-c | Reverse primer | TTCTGACAGCGGTGGAAGTC | 55.00 |
| GPx | Forward primer | CATCCTGCCTTCTGTCCCTG | 60.11 |
| GPx | Reverse primer | CGCCATGGCAGTCTGTCTTA | 60.11 |
| UCP2 | Forward primer | AGCCCACGGATGTGGTAAAG | 60.04 |
| UCP2 | Reverse primer | AGCCCACGGATGTGGTAAAG | 60.11 |
| GAPDH | Forward primer | GAAAGCCTGCCGGTGACTAA | 60.32 |
| GAPDH | Reverse primer | GATCTCGCTCCTGGAAGATGG | 60.00 |
