# Supplementary figures and images for: Clarithromycin synergizes with cisplatin to inhibit ovarian cancer growth in vitro and in vivo
Source: J Ovarian Res. 2019 Nov 8;12:107. doi: 10.1186/s13048-019-0570-9 (PMC6839134; doi:10.1186/s13048-019-0570-9)

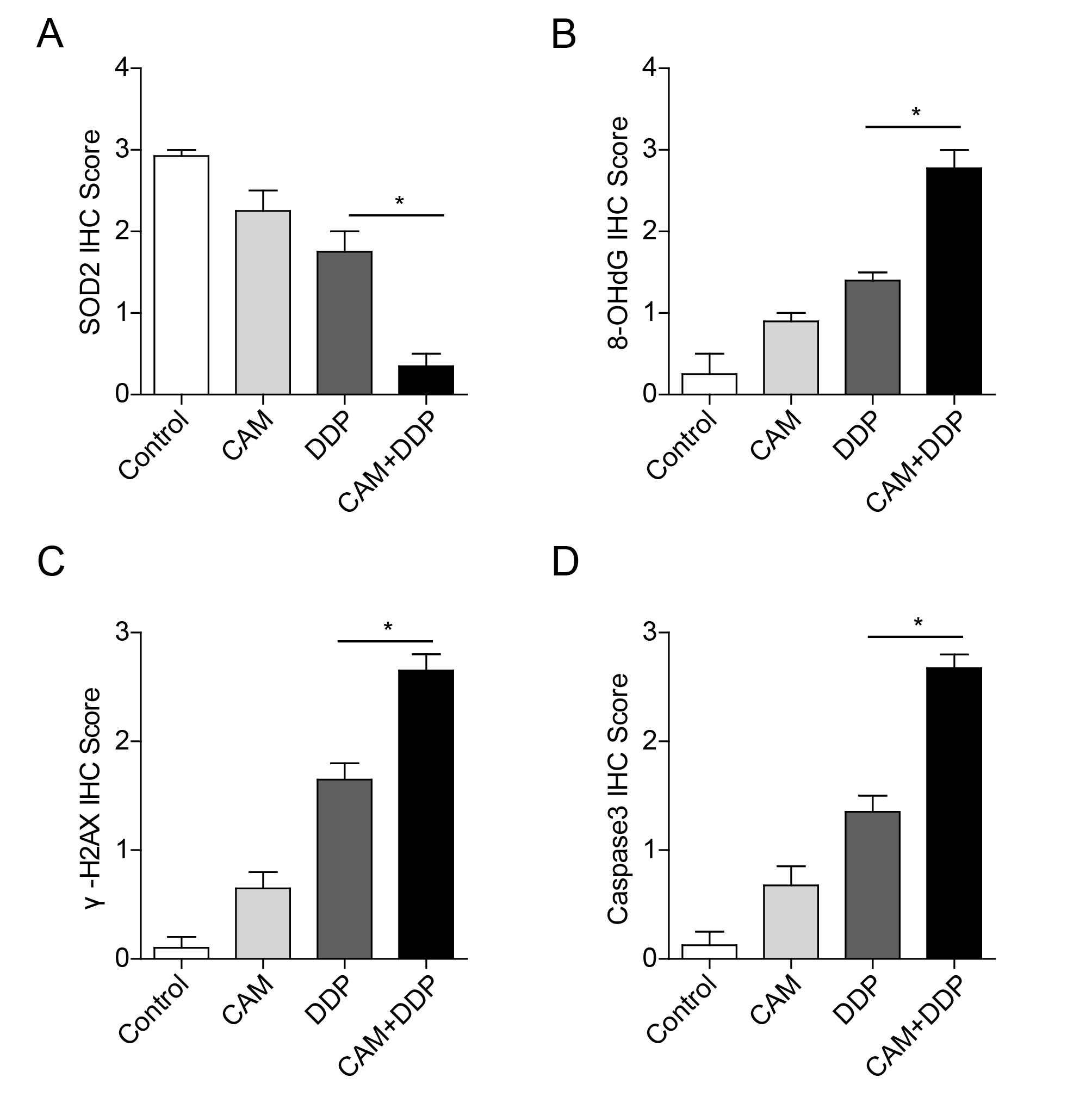

Supplement: Supplementary file 2 — Additional file 2. The IHC score of each gene is calculated as described in materials and methods (Fig. A,B,C and D). (TIF 461 kb) [file 13048_2019_570_MOESM2_ESM.tif]
